# Human Cerebral Organoid Implantation Alleviated the Neurological Deficits of Traumatic Brain Injury in Mice

**DOI:** 10.1155/2021/6338722

**Published:** 2021-11-22

**Authors:** Zhongyuan Bao, Kaiheng Fang, Zong Miao, Chong Li, Chaojuan Yang, Qiang Yu, Chen Zhang, Zengli Miao, Yan Liu, Jing Ji

**Affiliations:** ^1^Department of Neurosurgery, The First Affiliated Hospital of Nanjing Medical University, Nanjing, China; ^2^Department of Neurosurgery, The Affiliated Wuxi No. 2 People's Hospital of Nanjing Medical University, Wuxi, China; ^3^Institute for Stem Cell and Neural Regeneration, School of Pharmacy, Nanjing Medical University, Nanjing, China; ^4^School of Basic Medical Sciences, Beijing Key Laboratory of Neural Regeneration and Repair, Advanced Innovation Center for Human Brain Protection, Capital Medical University, Beijing, China; ^5^Department of Neurosurgery, Zhenjiang First People's Hospital, Zhenjiang, China

## Abstract

Traumatic brain injury (TBI) causes a high rate of mortality and disability, and its treatment is still limited. Loss of neurons in damaged area is hardly rescued by relative molecular therapies. Based on its disease characteristics, we transplanted human embryonic stem cell- (hESC-) derived cerebral organoids in the brain lesions of controlled cortical impact- (CCI-) modeled severe combined immunodeficient (SCID) mice. Grafted organoids survived and differentiated in CCI-induced lesion pools in mouse cortical tissue. Implanted cerebral organoids differentiated into various types of neuronal cells, extended long projections, and showed spontaneous action, as indicated by electromyographic activity in the grafts. Induced vascularization and reduced glial scar were also found after organoid implantation, suggesting grafting could improve local situation and promote neural repair. More importantly, the CCI mice's spatial learning and memory improved after organoid grafting. These findings suggest that cerebral organoid implanted in lesion sites differentiates into cortical neurons, forms long projections, and reverses deficits in spatial learning and memory, a potential therapeutic avenue for TBI.

## 1. Introduction

Traumatic brain injury (TBI) is one of the leading causes of death of young people in the developed world [1, 2]. TBI induces damage in two stages: an acute mechanical tissue lesion frequently leading to neuronal death and a chronic neuronal damage characteristic of neural degeneration in the contusive area [3, 4]. Presently, severe TBI is currently managed in the intensive care unit with a combined medical-surgical approach [5, 6]. Treatment aims to prevent additional brain damage and to optimise conditions for brain recovery [4]. However, neuron loss and limited regeneration at lesion sites cannot be redeemed via current some targeted molecular therapies. Stem cell therapy is a potential approach to enhance neuron repair capacity and has been pursued in almost 900 clinical trials worldwide (https://www.clinicaltrials.gov/ct2/home). Earlier studies have indicated that stem cell-transplantation therapy could combat various diseases, such as renal injury [7, 8], hematological disease [9, 10], and myocardial ischemia [11, 12]. In brain injury, human embryonic stem cell-derived neural stem cell (NSC) transplantation is a potential therapy for ischemic stroke by improving functional recovery [13, 14]. NSC transplantation has also been an probable effective therapy for TBI [15]. However, general NSC transplanting has shown poor survival and dissatisfactory differentiation [16], as scattered cells have not been able to gather in the damaged area without flowing away. Therefore, a modified method is necessary to improve the NSC development and differentiation *in vivo*.

We used three-dimensional (3D) neural tissues generated from human embryonic stem cells (hESCs), also termed “cerebral organoids,” which have previously been used in not only drug screening but also mechanisms of neurodevelopment exploration [17–19]. They have enormous potential to fill damaged areas and investigate neuroprotective responses because of their special characteristics: stable morphology and neural differentiation. Unlike general NSC therapy [20], we used cerebral organoids *in vitro* and implanted them. A spherical rosette structure was easy to transfer by tweezer and graft into lesion areas, the first use of human organoids in a SCID controlled cortical impact (CCI) mouse model.

We employed an efficient *in vivo* organoid engraftment model in CCI mouse brains. After a roughly 2-month implantation, micromagnetic resonance imaging (MRI) showed that implanted organoid had integrated into the surrounding mouse brain in the lesion pool. The grafted cells also exhibited neural differentiation and decent electrophysiological activities. Vascularization labeled by CD31 was also found, something never seen before in cerebral organoids *in vitro*. Reduced glial scar was found after about 60-day development after grafting, which could alleviate the impediment to axon growth and regeneration. Cerebral organoid implantation also improved the memory and spatial-learning abilities of mice after CCI. In summary, cerebral organoid transplantation is a potential therapy to reverse posttraumatic neuron loss and improve learning and memory ability.

## 2. Materials and Methods

### 2.1. Cerebral Organoid Culture and Transfection

Cerebral organoids were cultured from human pluripotent stem cells (hESCs) dissociated by dispase. The dispersed cells were suspension-cultured in a flask to generate embryonic bodies (EBs) with neural induction medium (NIM), which contained DMEM/F12, 1% N2 supplement, and 1% MEM-NEAA. On day 7 (D7), EBs were attached to dishes for neural tube formation. On D16, cells were resuspended by pipette to form neurospheres (NSs), which were transferred into Matrigel droplets one by one and further cultured in a 60 mm dish on D25, finally forming cerebral organoids. All information of used reagents is shown in Supplementary Table 1. In this experiment, the cultured organoid needed to express GFP in subsequent experiments. In brief, cerebral organoids were transfected with the lentivirus carrying GFP (Genechem, Shanghai, China) and were transfected to up to 20 MOI with 10% polybrene in a 60 mm dish. After 12 h incubation at 37°C, the neural-basal medium was changed with a fresh one. And green fluorescence intensity was obtained using a Nikon Eclipse E600 microscope (Nikon, Melville, New York).

### 2.2. Immunofluorescent Staining

In brief, thirty-five-micrometer frozen sections were fixed by 4% paraformaldehyde for 1 h and then incubated in phosphate-buffered saline (PBS) with 0.3% Triton X-100 and 5% bovine serum albumin for 1 h. After blocking, sections were stained with relative antibody at 4°C for 8 h or overnight. Subsequently, secondary antibodies (Alexa Fluor, 549 or 488; Invitrogen) were used, and Hoechst (Ho) was used to visualize cell nuclei. Images were obtained using a Nikon Eclipse E600 microscope (Nikon, Melville, New York). All information of used primary antibodies was shown as followed: Rabbit polyclonal anti-Doublecortin (DCX), Abcam, Cat# ab18723; Mouse polyclonal antibeta III Tubulin (Tuj-1), Abcam, Cat# ab18207; Rabbit polyclonal anti-Tbr1, Santa Cruz Biotechnology, Cat# sc-48816; Rabbit polyclonal anti-Tbr2, Abcam,Cat# ab23345; Rabbit polyclonal anti-Ctip2, Abcam, Cat# ab18465; Rabbit polyclonal anti-Foxp2, Abcam, Cat# ab16046; Rabbit polyclonal anti-Pax6, Abcam, Cat# ab195045; Rabbit polyclonal anti-Nanog, Cell Signaling Technology, Cat# 4903; Rabbit polyclonal anti-Sox2, Abcam, Cat# ab93689; Rabbit polyclonal anti-Ki67, Abcam, Cat# ab15580; Mouse polyclonal anti-Brn2, Millipore, Cat# MABD51; Mouse polyclonal anti-Stab2, Abcam, Cat# ab51502; Rabbit polyclonal anti-Hopx, (Santa Cruz Biotechnology, Cat# sc-30216; Rabbit polyclonal anti-CD31, Abcam, Cat# ab28364; Mouse monoclonal anti-human nuclei, Millipore, Cat# MAB1281; and Mouse monoclonal anti-STEM121, Takara Bio, Cat# Y40410.

Organoids seeded onto a glass wafer were fixed with cooled 4% paraformaldehyde for 15 min and incubated in PBS with 0.1% Triton X-100 and 5% bovine serum albumin for 1 h. The following processes were same as those in tissue staining.

### 2.3. Animal and Implantation Model

All experimental procedures performed in SCID mice were preapproved and performed according to guidelines established by the Nanjing Medical University Administrative Panel on Laboratory Animal Care. SCID mice for all experiments were female and were randomly assigned to experimental groups. Eight-week-old male SCID mice (Nanjing Medical University, Nanjing, China) were subjected to severe CCI as previously described [21]. Anesthesia was induced with 3% isoflurane (R.W.D., Shenzhen, China) in nitrous oxide : oxygen (7 : 3) and maintained with 1.5% isoflurane via a nose cone. After anesthesia, the mice were fixed to a stereotaxic frame (R.W.D., Shenzhen, China). The skin was cut open, and the bone with a diameter of 5 mm overlaying the left parietal cortex was removed with a drill. CCIs were produced using an impactor tip (6.0 ± 0.2 m/s, 50 ms dwell time, 1.4 mm depth). The scalp was sutured, and another 7 days passed before cutting the scalp again and implanting organoids. A small crevasse was made in the subcutaneous soft tissue, and organoids were grafted into the lesion area by tweezer. Notably, the crevasse in the subcutaneous soft tissue was suitable for tweezers to reach the lesion pool; an oversized crevasse might have influenced the success rate of transplantation because cerebrospinal fluid outflow could have pushed out the grafted organoids. The scalp was sutured again, and the temperature of mice was maintained with a heating pad.

### 2.4. Morris Water Maze Test

All experiments were performed in a circular pool (2.0 m in diameter) with a light approximately 2.0 m above its center and 4 distinct labels on the wall of the pool. The average of the data in the 4 quadrants is the datum of the sample. Water temperature was maintained at 22 ± 1°C and was made opaque using dark paint during hidden training-session tests.

First, mice (*n* = 8 mice in sham and *n* = 10 in other groups) were individually handled for 1 day before starting the experiment to acclimate them to introduction to and removal from the pool. After the habituation period, mice were subjected to 3-day visible training sessions (4 trials per day) in which a platform (10 cm in diameter) was made visible by attaching a light-colored cubic landmark. If the mouse found the platform within 1 min, it remained there for 5 sec. If not, it was gently guided toward the platform before staying on it. After the 5 s period on the platform, the mice were dried on a heating pad and then transported back to their cage.

Following the visible training sessions, the mice were subjected to 7-day hidden training sessions (4 trials per day) in which the platform was placed 1.0 cm below the water surface. Platform's location was fixed throughout the experiment. If the mice found the platform, the data was recorded. When mice could not find the platform within 60 s, they were guided toward it and stayed on it for 10 s, and the latency to reach the platform was recorded as 60 s. The starting position was random in each quadrant trial. For each trial, the latency to reach the platform(s), swimming distance (cm), and swimming speed (cm/s) were measured using Time MWM software (O'Hara & Co., Tokyo, Japan).

### 2.5. Micro-MRI Assay

An MRI apparatus for mice at 11.7 T (Bruker, AVANCE 500 WB) offered by the Animal Core Facility of Nanjing Medical University was used for the experiments. The mice were anesthetized with 3% isoflurane and maintained with 1.5% isoflurane via a nose cone. MRI was performed under the following conditions: a T2-weighted fast spin echo (RARE) sequence (TR/TE = 5000/35.4 ms; FOV = 25 × 25 mm; matrix size = 256 × 256; slice thickness = 0.5 mm; NS = 8; total scan time = 10 min approx.).

### 2.6. Passive Avoidance Assay

The mice in all groups were put in the light side of a light-dark box on the first day (D1), although they tended to wander in the dark rooms. On D2 and D3, 0.3 mA of electricity stimulation was applied in the dark box, forcing all the mice to stay on the light side. On the fourth day, the mice were put in a light-dark box without electricity stimulation on the dark side. Motion curves and duration in light and dark box on the first and fourth days were recorded by software.

### 2.7. Electrophysiological Test

Recordings were performed with mice that had been grafted for 70 days. The mice were deeply anesthetized and underwent infusion with cold 95% O_2_ and 5% CO_2_ saturated sucrose-substituted artificial cerebrospinal fluid (aCSF) via the left ventricle. The aCSF contained the following (in mM): 110 mM of sucrose, 60 mM of NaCl, 3 mM of KCl, 1.25 mM of NaH_2_PO_4_, 28 mM of NaHCO_3_, 5 mM of D-glucose, 500 *μ*M of CaCl_2_, 7 mM of MgCl_2_, and 600 of *μ*M ascorbate. Coronal slices 400 *μ*m thick were prepared with a Vibratome and incubated in identifiable positions on a mesh in oxygenated aCSF for at least 1 h before electrophysiological recording. The slices were then transferred to a recording chamber and perfused (1 ml/min) with 100% aCSF. All solutions were continuously bubbled with 95% O_2_ and 5% CO_2_. Grafted organoids were found using 488 fluorescence-excitation spectra. Spontaneous potentials were recorded and action potentials acquired using suction electrodes for both stimulating and recording, as previously described [22, 23]. All potentials were recorded with a HEKA amplifier. The signals were processed using Metalab (PatchMaster) software.

### 2.8. Statistical Analyses

The data were analyzed with GraphPad 8.0 software and reported as the mean ± standard deviation. An unpaired *t*-test or one-way analysis of variance (ANOVA) and Tukey's test were applied to relative immunofluorescence intensity measures and a behavior test at each time point including 61 to 70 dpi to compare the differences between the two groups. For behavioral tests, including the Morris water maze (MWM) assays, the data of the whole group were analyzed using two-way ANOVA with repeated measures followed by Tukey *post hoc*. Significant differences were set at *P* < 0.05.

## 3. Results

### 3.1. Generation of Human Cerebral Organoids Cultured In Vitro

**Figure 1:**
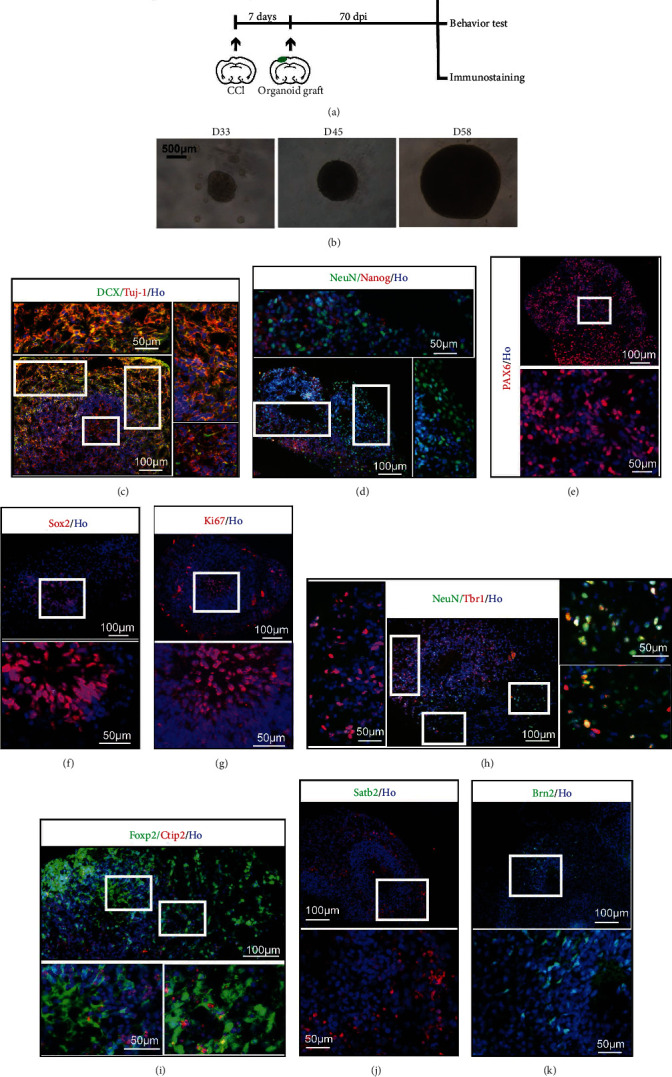
Human cerebral organoids cultured *in vitro*. (a) Experimental procedure of this study including cerebral organoid implantation and schedule. (b) Generation of organoids from human ESCs. The images were taken at 33 days, 45 days, and 58 days. (c) Immunohistochemistry for neurons (Tuj-1, red) and the new development of the cerebral cortex (DCX, green). Scale bars are 100 *μ*m and 50 *μ*m. (d) Staining for mature neurons (NeuN, green) and embryonic stem cell (Nanog, red). Scale bars are 100 *μ*m and 50 *μ*m. (e) Immunohistochemistry in the section for the forebrain marker Pax6. Scale bars are 100 *μ*m and 50 *μ*m. (f) Staining for embryonic stem cell pluripotency (Sox2, red). Scale bars are 100 *μ*m and 50 *μ*m. (g) Staining for the cell proliferation marker Ki67. Scale bars are 100 *μ*m and 50 *μ*m. (h) Staining for the preplate marker Tbr1 (red) and neuronal marker NeuN (green) revealing organoid maturity. Scale bars are 100 *μ*m and 50 *μ*m. (i) Staining for deep-layer subcortical neuron marker Ctip2 (red) and developing (Foxp2, green) neurons. Scale bars are 100 *μ*m and 50 *μ*m. (j) Staining for Satb2 (red). Scale bars are 100 *μ*m and 50 *μ*m. (k) Staining for Brn2 (green). Scale bars are 100 *μ*m and 50 *μ*m. All cell nuclei were stained by Hoechst (Ho).

An experimental process was shown (Figure 1(a)). In brief, before organoid implantation, controlled cortical injury (CCI) was performed at 7 days in advance. 2 months approximately were needed for grafted organoid to differentiate, mature, and develop. Human ESC-derived organoids were cultured *in vitro*, their sizes increasing over time (Figure 1(b)). Images of the organoids on the 33^rd^, 45^th^, and 58^th^ days under 3D culture were displayed. Organoids efficiently expressed the characteristics of newborn neurons labelled by DCX and Tuj-1 on day 58 (Figure 1(c)). Dcx is a microtubule-associated protein required for initial steps of neuronal dispersion and cortex lamination during cerebral cortex development. It participates in a signaling pathway that is crucial for neuronal interaction before and during migration, possibly as part of a calcium ion-dependent signal transduction pathway [24, 25]. Expression of Tuj-1 is primarily restricted to central and peripheral nervous system. Tuj-1 plays a critical role in proper axon guidance and maintenance, which is a pan-neuronal marker [26, 27]. At this time point, mature neurons (labeled “NeuN”) and neural stem cells (labeled “Nanog”) were determined in the organoids (Figure 1(d)). Pax6, a marker of neural precursor cells and expressed in the developing central nervous system [28, 29], was also detected (Figure 1(e)). Cells in the center lumen expressed a neural stem cell marker (labeled “Sox2”) and a cell proliferation marker (labeled “Ki67”) (Figures 1(e) and 1(g)). The 58-day 3D culture conducted differentiated stem cells to Tbr1-positive cells (Figure 1(h)). Tbr1 is a characteristic of the deep cortical layer [30] and a transcriptional repressor involved in multiple aspects of cortical development, including neuronal migration, laminar and a real identity, and axonal projection [31, 32]. Besides Tbr1, the expressions of Ctip2 and Foxp2 were detected (Figure 1(i)). In general, Ctip2 is expressed in many brain regions, but its expression is prominent in corticospinal motor neurons (CSMNs) [33]. The transcription factor Foxp2 is involved in setting up the neuronal circuitry for vocal learning in mammals and is thought to have played a special role in the evolution of human speech and language [34]. And it is also associated with inherited dyspraxia and poor control of the facial musculature [35]. Satb2, an up-layer marker [36], was detected at this time point as well (Figure 1(j)). During embryonic development, Satb2 is essential for the establishment of the proper identity and axon projections of callosal neurons in mouse neocortex [37]. Brn2, a neuron-specific transcription factor [38], was also found in 58-day cerebral organoids (Figure 1(k)). It is an essential transcription factor in neocortical neurogenesis [39]. Overexpression of Brn2 enhanced neuronal differentiation and attenuated astrocyte differentiation in neocortical multipotent neural precursor cells (NPCs) cultures [40]. Therefore, cerebral organoids undergoing 3D culture exhibited the identities of cortical layers and neural progenitor cells (NPCs).

### 3.2. Implanted Organoids Displayed Appropriate Growth and Development

**Figure 2:**
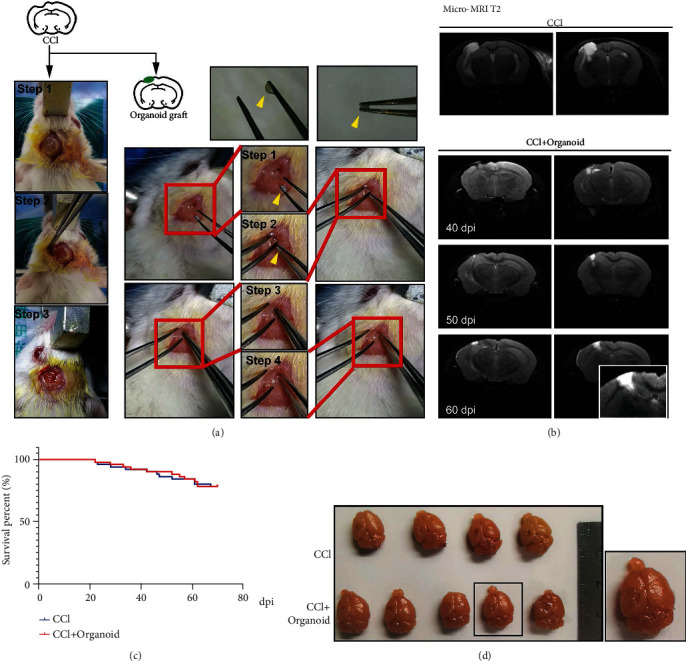
Organoids grafted into SCID CCI-model mice exhibited appropriated growth and survival. (a) Schematic image of CCI (3 steps) and organoid graft (4 steps). Organoids could be picked up by tweezers (yellow arrow). (b) Micro-MRI T2 assay was used to display tissue lesions in CCI mice and the growth situation of grafted organoids at 40 dpi, 50 dpi, and 60 dpi. (c) Survival percentage of SCID mice in CCI group and CCI+organoid group. (d) Image of mouse brain tissues in the CCI and CCI plus organoid groups.

Distinct from previous method to inject neural stem cells into mouse brain [41], 58-day organoids were implanted by tweezer. A brief process of operation is displayed in Figure 2(a). The controlled cortical impact (CCI) was performed in SCID mice, and subsequent 7 days were needed to avoid the local inflammatory storm. A small crevasse was made in the subcutaneous soft tissue, and organoids were grafted into the lesion area by tweezer. Notably, the size of crevasse in the subcutaneous soft tissue was suitable for tweezers to reach the lesion pool. In order to detect the lesion after CCI injury and the development of graft organoids, a micro-MRI test was used. The cerebrospinal fluid (CSF) filled in the lesion site. The statuses of grafted organoids at 40 days postimplantation (dpi), 50 dpi, and 60 dpi are also shown in Figure 1(b). As the dpi increased, the grafted organoids gradually grew in the lesion area. Especially, implanted organoid grew well and had a tend to extend at 60 dpi. 75% of the grafted animals survived beyond 70 dpi, which presented no difference than CCI group (Figure 2(c)). Morphologically, implanted organoid could fill up CCI-induced lesion pool, but not grow like an intracranial tumor occupying (Figure 2(d)). Therefore, it is appropriate that mouse brains grafted with cerebral organoids were harvested at 55–70 dpi and analyzed, on the basis of which an approximately 2-month implantation period was used for further study.

### 3.3. Implanted Organoids Exhibited Progressive Differentiation

**Figure 3:**
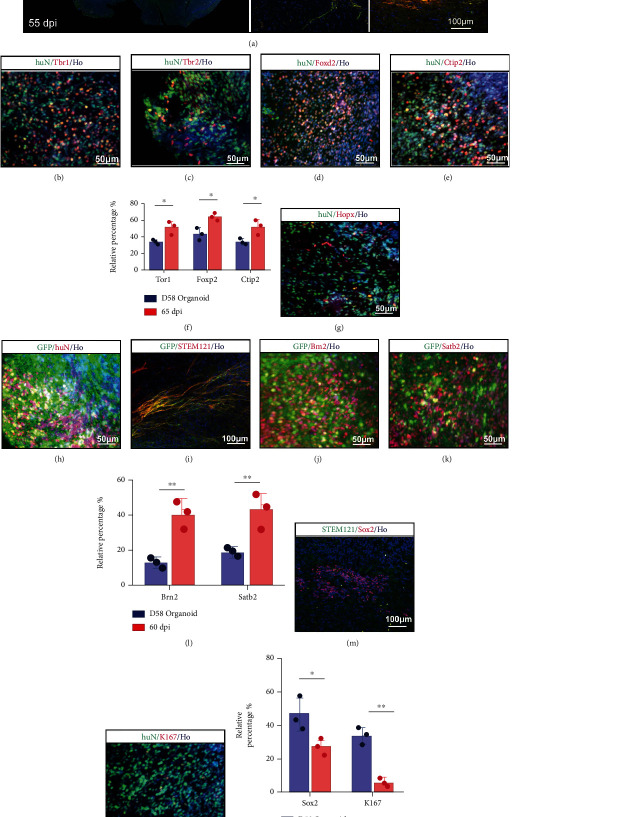
Degree of differentiation and maturity of grafted human cerebral organoids. (a) Immunohistochemistry for human-derived cells (STEM121, green) and the new development of neuronal cells (DCX, red). The grafted cells could migrate to the contralateral brain region via the callosum. Scale bar is 200 *μ*m. (b, c) Staining for the preplate marker Tbr1 (red) and intermediate progenitor marker Tbr2 (red). Grafted human-derived cells were both stained for huN (green). Scale bars are 50 *μ*m. (d, e) Staining for developing neuronal cells (Foxp2, red) and early-born neurons (Ctip2, red). Grafted human-derived cells were both stained for huN (green). Scale bars are 50 *μ*m. (f) Compared to organoids *in vitro*, the percentage of grafted neurons expressing Tbr1, Foxp2, and Ctip2 increased. Error bars represent S.D. (*n* = 3 experiments). ^∗^*P* < 0.05 versus relative organoid group *in vitro*. (g) Staining for neural progenitor (Hopx, red) involved in differentiation of neurons in OSVZ. Grafted human-derived cells were stained for huN (green). Scale bar is 50 *μ*m. (h, i) Development of organoid conjunct with GFP. (h) Staining for GFP (green) and huN (red). Scale bar is 50 *μ*m. (i) Staining for GFP (green) and STEM121 (red). Scale bar is 100 *μ*m. (j) Staining for neuronal subtype progression in cortex (Brn2, red). GFP was used to label grafted cells. Scale bar is 50 *μ*m. (k) Staining for late-born neurons (Satb2, red). GFP was used to label grafted cells. Scale bar is 50 *μ*m. (l) Compared to organoids *in vitro*, the percentage of grafted neurons expressing Brn2 and Stab2 increased. Error bars represent S.D. (*n* = 3 experiments). ^∗∗^*P* < 0.01 versus relative organoid group *in vitro*. (m) Staining for embryonic stem cell pluripotency (Sox2, red). Scale bar is 100 *μ*m. STEM121 was used to display human-derived cells. (n) Staining for Ki67 to reveal cell proliferation level. huN was used to display human-derived cells. Scale bar is 50 *μ*m. (o) The percentage of grafted neurons expressing Sox2 or Ki67 decreased in organoids 65 dpi. Error bars represent S.D. (*n* = 3 experiments). ^∗^*P* < 0.05 or ^∗∗^*P* < 0.01 versus relative organoid group *in vitro*. All cell nuclei were stained by Hoechst.

Before implantation, 40 organoids with GFP and 65 organoids without GFP were cultured for further experimental processes. To examine whether progressive differentiation and maturation occurred over time *in vivo*, we examined the newborn neural marker Doublecortin (DCX) and the human-specific cytoplasmic marker STEM121, indicating developing, well-differentiated neural cells, even with projection to the other hemisphere via the corpus callosum at 55 dpi (Figure 3(a)). At 65 dpi, implanted organoids expressed Tbr1, Tbr2, Foxp2, and Ctip2 (Figures 3(b)–3(e)). Compared to the 58-day cerebral organoids *in vitro*, a higher percentage of cells expressing Tbr1, Foxp2, and Ctip2 was found at 65 dpi *in vivo*, suggesting that the *in vivo* environment enhanced cellular maturation and cortical differentiation (Figure 3(f)). Not only were cortex markers detected but also Hopx, which represents the outer subventricular zone (OSVZ) area (Figure 1(g)). Hopx expression distinguishes between adult neural progenitors in the SGZ and SVZ and potentially regulates dentate neurogenesis [42]. The GFP-conjunct organoids were also grafted into CCI-model mice and survived well (Figures 3(h) and 3(i)). Brn2 was highly expressed two months after implantation (Figure 3(j)). Satb2, which regulates the differentiation of both callosal and subcerebral projection neurons in the developing cerebral cortex [37], was detected in transplanted organoids at 60 dpi (Figure 3(k)). Compared to cerebral organoids *in vitro*, the numbers of Brn2^+^ and Stab2^+^ cells increased at 60 dpi over those *in vitro* (D58) (Figure 3(l)). At their stage of *in vitro* culture, cerebral organoids consisted of both NPCs and mature neuronal cells (labeled “NeuN”). To determine whether similar progressive maturation occurred over time *in vivo*, we sought Sox2 and Ki67 expression (Figures 3(m) and 3(n)). At 65 dpi, Sox2 and Ki67 expression was observed in grafted organoids but less so than in cerebral organoids *in vitro* (Figure 3(o)). Altogether, grafted organoids exhibited progressive differentiation *in vivo*.

### 3.4. Implanted Organoid Development Contributes to Local Neural Repair

**Figure 4:**
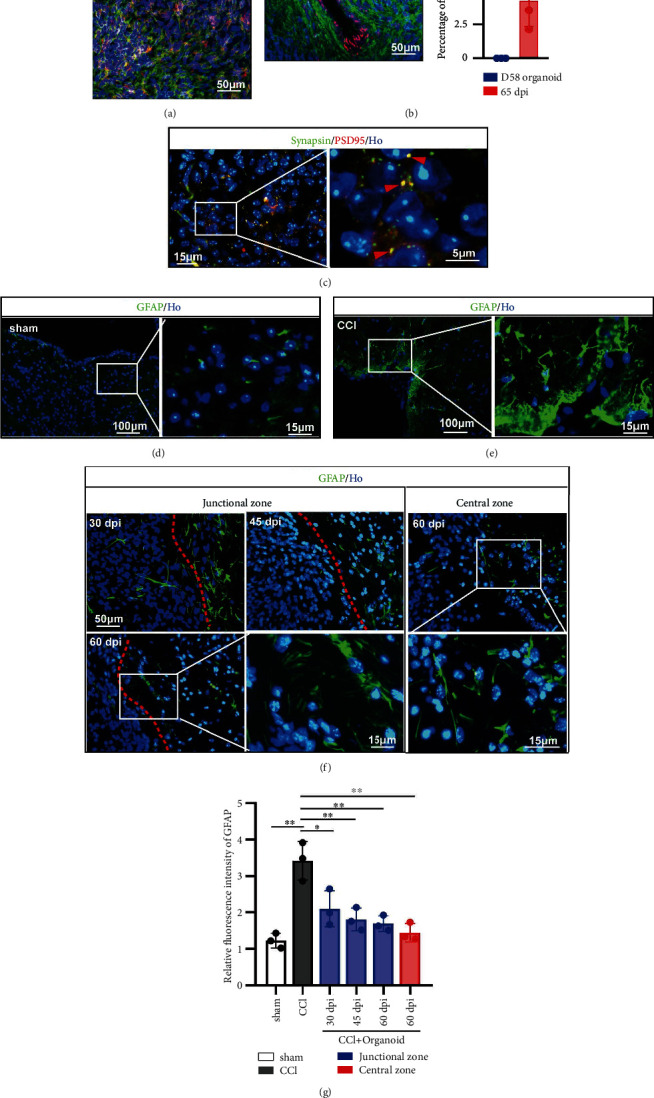
Grafted organoid improved local situation contributing to neural repair. (a) Staining for glutamate (red). Grafted human-derived cells were stained for STEM121 (green). Scale bar is 50 *μ*m. (b) Vascular endothelial cell was labeled by CD31 (red). Grafted human-derived cells were stained for STEM121 (green). Scale bar is 50 *μ*m. All cell nuclei were stained by Hoechst (Ho). The percentage of CD31 was displayed in the bar graph (right part). (c) Double immunofluorescence staining for presynaptic marker Synapsin and the postsynaptic marker PSD95 at 60 dpi, showing a coassociation between pre- and postsynaptic compartments and the formation of synaptic connections in the graft. Scale bar is 15 *μ*m and 5 *μ*m. (d, e) The formations of glial scar in sham and CCI groups were detected by staining for GFAP. Scale bar is 100 *μ*m and 15 *μ*m. (f) In grafted model, GFAP was detected in junctional (left) and central zone (right). GFAP in junctional zone was recorded in 30 dpi, 45 dpi, and 60 dpi. Scale bar is 50 *μ*m and 15 *μ*m. GFAP in central zone was displayed in 60 dpi. Scale bar is 50 *μ*m and 15 *μ*m. (g) Compared to sham group, GFAP was significantly increased around lesion area. Grafted organoid alleviated the GFAP expression in junctional or central area, suggesting a reduced formation of glial scar. Error bars represent S.D. (*n* = 3 experiments). ^∗^*P* < 0.05 versus CCI group; ^∗^*P* < 0.05 versus sham or CCI group *in vivo*.

Besides detected differentiated process, improved local situation was also a pusher for neural repair. Glutamate is the major excitatory neurotransmitter in the central nervous system [43]. In grafted organoids, glutamate was also detected *in vivo* (Figure 4(a)). Vascularization is especially necessary for nutrient supply and efficient neural progenitor differentiation, which the cerebral organoid systems *in vitro* lacked but was detected by CD31 in grafted organoids *in vivo* (Figure 4(b)). The presynaptic marker Synapsin and the postsynaptic marker PSD95 were detected by immunostaining, showing multiple Synapsin and PSD95 colocalized puncta and suggesting synaptic connectivity in the grafts (Figure 4(c)). The formation of glial scar is one of important factors that affected regeneration of nerve fiber and neuron [44]. Abundant astrocytes labeled by GFAP, especially around injury lesion, were found after CCI (Figures 4(d) and 4(e)). Implanted organoids alleviated GFAP expression in the junctional area at 30 dpi, 45 dpi, and 60 dpi (Figure 4(f) left). In the central area of grafted organoid, GFAP was also found (Figure 4(f) right), whose fluorescence intensity was similar like sham group but lower than that of CCI group (Figure 4(g)). Therefore, grafted organoid contributes to improve local situation and promote neural repair.

### 3.5. Neuroprotective Effects of Mature Organoids with Neural Electrophysiological Activity and Animal Behavioral Improvements

**Figure 5:**
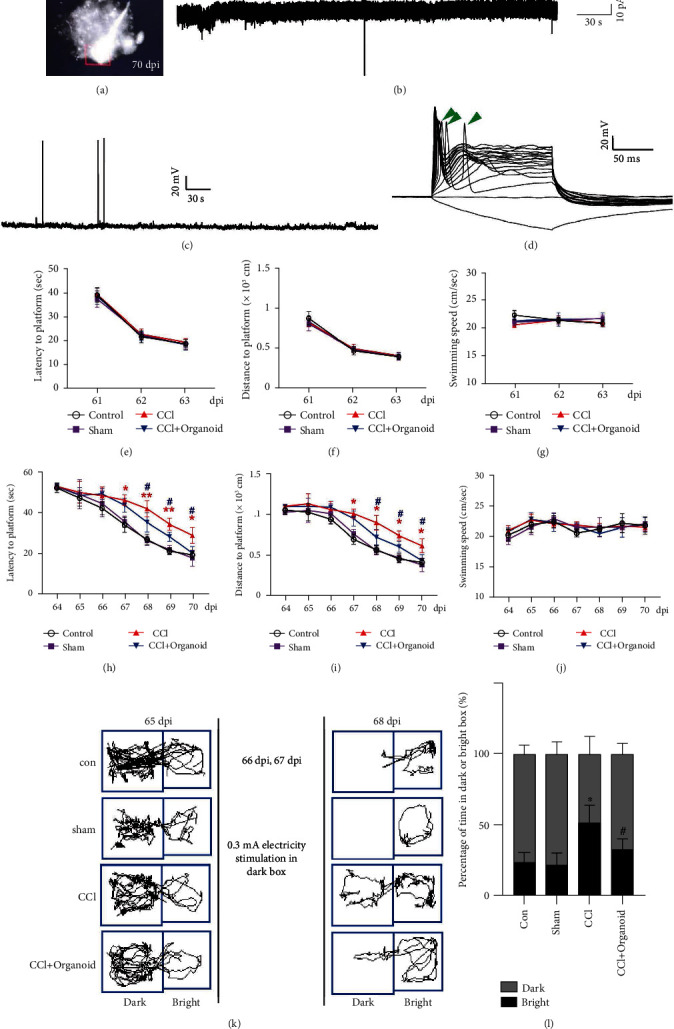
Neuroprotective effects of organoid implantation. (a) Cells carrying GFP were eligible for electrophysiological examination. (b) Spontaneous potential and (c, d) action potential were recorded. Green arrows indicate stimulated action. Training for mice to locate visible (e–g) or hidden (h–j) platforms 61–70 dpi is shown in Morris water maze. *n* = 8–10. Error bars represent s.d. (*n* = 8–10 mice per group). One-way ANOVA followed by Tukey's *post hoc* test was used to analyze the difference between groups at each time point (^∗^ or ^#^ labels the relative time points). ^∗^*P* < 0.05 and ^∗∗^*P* < 0.01 versus sham group; ^#^*P* < 0.05 versus CCI group. A two-way ANOVA with repeated measures followed by Tukey's *post hoc* test was used for the whole groups, revealing the group-by-day interaction effect in latency to platform (*F*_18,217_ = 6.405, *P* < 0.0001), swimming distance (*F*_18,21_ = 5.265, *P* = 0.0145), and swimming speed (*F*_18,217_ = 2.200, *P* = 0.0043) during the hidden test. (k, l) Passive avoidance assay was performed. Course and time in the dark and bright boxes were recorded and analyzed, respectively. Error bars represent s.d. (*n* = 8 mice per group). One-way ANOVA plus Tukey's test was used to measure the time in the bright box after electrical stimulation. ^∗^*P* < 0.05 versus sham group; ^#^*P* < 0.05 versus CCI group.

To determine whether organoid grafts exhibited electrophysiological activity, we conducted cellular recording using multielectrode arrays at 70 dpi. Cells expressing GFP were found (Figure 5(a)). We also successfully recorded spontaneous potentials (Figure 5(b)) and action potentials (Figures 5(c) and 5(d)) under stimulation. Thus, the grafted organoids might have differentiated into mature neural cells and been transmitting neural information. CCI-induced neural injury also includes chronic neurodegeneration, two characteristics of which are disabilities in memory and cognition. We conducted behavioral tests, including the Morris water maze (MWM) test and a passive avoidance assay. In the MWM test, mice were first subjected to performing 3-day visible training, during which the platform was on the surface of the water indicated by black staining. In the 3-day visible training session, no difference was found in latency, distance, or swimming speed to the platform, indicating that all the mice had no visual disorders and no differences in motor abilities (Figures 5(e)–5(g)). In the hidden test, mice with organoid grafting exhibited better spatial learning abilities than mice with only CCIs, with shorter latency and distances to the platform starting on the fifth hidden test day. Swimming speeds in this test did not change at any time, excluding the well-known interference of motor factors in whole-MWM experiments (Figures 5(h)–5(j)). Besides MWM, the passive avoidance assay was another test for short-term memory (Figures 5(k) and 5(l)). Electric stimulation in the dark box forced mice to stay in the light box despite their natural preference for the dark environment. On the test day, mice treated with organoid grafting still preferred to remain in light box more than CCI mice with the electric stimulation removed. Thus, grafted mice were more likely to remember the danger in the dark box and remain in the light box. Taken together, grafted organoids for about 2 months improved the learning and memory ability of CCI-induced mice.

## 4. Discussion

TBI induces irreversible neuron loss and results, not only in acute injury but also in chronic injury associated with neurodegenerative disorders characterized by memory deterioration. We introduced a novel method of implanting cerebral organoids into CCI-induced lesion areas. Unlike earlier studies about relative molecular targeting treatments reducing neuronal death in surrounding contused brain tissues [45, 46], the implantation of cerebral organoids, not general NSCs, efficiently filled up lesion pools and restored the functions of original neurons in lesion areas, surpassing the low survival rate and poor differentiation of NSC implantation. In contrast to general 2D NSC culture, 3D-cultured organoids generated structure characteristics of neurons, including relative human brain organogenesis and cytoarchitecture, and have been used as models for studies of relative brain development and diseases in previous studies [47, 48]. Implanting 3D-cultured organoids was also more convenient than implanting 2D-cultured NSCs. Previous NSC transplantations have been performed by cell suspension injection into local tissues [49], which is not efficient because scattered cells could not gather in CCI-induced brain lesions, presenting poor survival and differentiation rates. Cultured cerebral organoids also kept their spherical volumes *in vitro* and could be picked up by tweezers. In this study, the size of the 58-day cultured organoids was suitable for picking up without using a stereoscope.

Grafted organoids at 60 dpi increased the percentage of Tbr1, Foxp2, and Ctip2 over those *in vitro*, revealing that more differentiated and matured cortical neural cells were generated *in vivo*. Increased Brn2^+^ and Satb2^+^ neural cells also indicated further differentiation in a specific direction. Sox2^+^ and Ki67^+^ cells were dramatically reduced, however, suggesting that the cells differentiated rather than keeping their original proliferation capacity. When cerebral organoids were cultured *in vitro*, their increasing size might have induced necrosis in their centers due to poor oxygen penetration and nutrient supply. Vascularization labeled by CD31 was also detected *in vivo*, supplying essential assistance for differentiation and maturation, and no necrotic cells were found in the grafted organoids, although this was investigated by an earlier study in which the vessels originated from the host [16]. Beside of vascularization, the formation of glial scar is another significant factor that impacts neural repair. CCI-induced glial scar expressing abundant GFAP could inhibit axon generation, which blocks organoid differentiation [50]. After organoid implanting, junctional area between organoid and the host brain tissues also displayed decreased GFAP expression. Grafted organoids grew in the lesion area and induced differentiation to long neural projection, which was due to the reduction in glial scarring. Therefore, organoid implantation improved local situation benefit to cell differentiation and mature. In the central of the grafted organoid, GFAP was also detected but not aggressive like CCI-induced change. Previous studies indicated that an appropriated astrocyte or level of GFAP was essential to neural differentiation [51, 52]. Therefore, improved situation by grafted organoid was a pusher to promote cell differentiation.

In the present study, spatial-learning ability was detected through MWM assays, indicating that grafted organoids had alleviated functional memory impairment. One previous study suggested that cerebral organoid transplantation also improves neurological motor functions in rats after brain injury, but their experimental design differed from ours in key ways [53]. They transplanted human organoids into Sprague Dawley rat brains and used cyclosporin A to inhibit immunological rejection. However, motor deficiency, which is generally tested soon after brain injury, can be compensatorily recovered over time without therapy; testing it 2 months postimplantation might be inappropriate for finding motor-ability differences between groups. Our study also found no difference between the swimming speeds of the groups by MWM assay, establishing a control to test their spatial learning abilities. In summary, organoids grafted for almost two months can adapt ontological brain tissues in developmental, structural, and functional aspects.

As for the appropriate implantation time, we chose the seventh day after CCI. Unlike other central nervous system (CNS) disease models, such as Alzheimer's disease and Parkinson's disease, CCI-induced acute injury combined with bleeding is inconvenient for transplanting; immediate implanting after CCI is thus inefficient. Plenty of bleeding inside lesions after CCI might carry away the organoids, effectively changing the grafting location. Rapid inflammatory responses after CCI would also influence the direction of differentiation and maturation, and greater TNF-*α* expression might inhibit the differentiation and proliferation of neural stem cells by upregulating Acsl2 [54]. CCI-induced microglia activation also promoted the expression of IL-1*β*, IL-6, and IL-18, affecting and interfering with neurogenesis [55, 56]. The enhanced inflammatory response still persisted on the seventh day after CCI, but not as explosively as the initial period. Therefore, avoiding a rapid inflammatory storm after CCI is necessary for cerebral organoid implantation.

## 5. Conclusions

**Figure 6:**
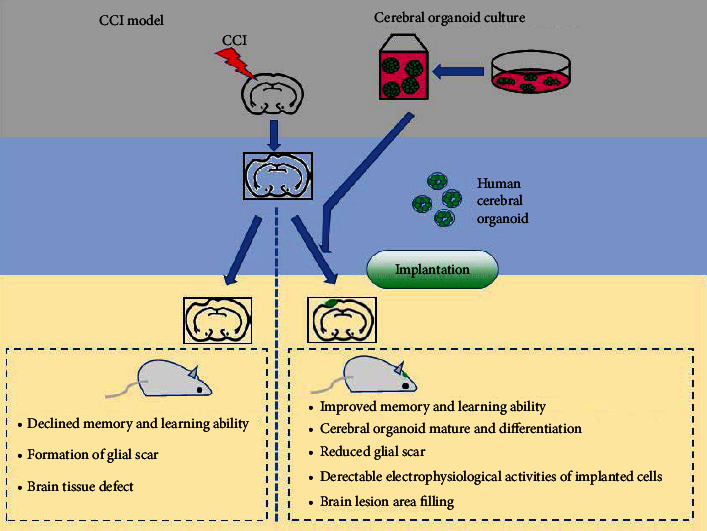
A schematic diagram showing the neuroprotective effects of human cerebral organoid implantation.

In summary, our study suggested that the enhanced differentiation and maturation of human cerebral organoids implanted in lesioned mouse cortices with good host-brain integration provides a novel cell-replacement strategy for addressing TBI and other neurodegenerative disorders (Figure 6). These observations also point to future directions of enquiry. For instance, how to improve the survival rate of neural stem cell in the brain of the host? If so, does the use of cerebral of organoid solve this problem? Also, is it more convenient to use organoid transplantation? Finally, whether organoid graft can relieve glial scarring caused by traumatic brain injury? If so, improved local condition would promote the communication of neural cells and functional reconstruction. Answers to these questions will offer valuable insights into the structural and functional effects triggered by traumatic brain injury, which might show clinically effective therapeutic interventions.

## Data Availability

The datasets generated during the current study are available from the corresponding authors upon reasonable requests.

## Supplementary Materials

Supplementary Materials The source, Cat# number and RRID of all antibodies and chemicals are shown.
