# Supplementary material for: Human Cerebral Organoid Implantation Alleviated the Neurological Deficits of Traumatic Brain Injury in Mice
Source: Oxid Med Cell Longev. 2021 Nov 22;2021:6338722. doi: 10.1155/2021/6338722 (PMC8629662; doi:10.1155/2021/6338722)
Supplement: Supplementary Materials — The source, Cat# number and RRID of all antibodies and chemicals are shown. [file 6338722.f1.docx]

**Supplementary Table 1**

| REAGENT | SOURCE | IDENTIFIER |
| --- | --- | --- |
| **Antibodies** |  |  |
| Rabbit polyclonal anti-Doublecortin (DCX) | Abcam | Cat# ab18723,  RRID: AB_732011 |
| Mouse polyclonal anti-beta III Tubulin (Tuj-1) | Abcam | Cat# ab18207,  RRID: AB_444319 |
| Rabbit polyclonal anti-Tbr1 | Santa Cruz Biotechnology | Cat# sc-48816, RRID: AB_2287060 |
| Rabbit polyclonal anti-Tbr2 | Abcam | Cat# ab23345,  RRID: AB_778267 |
| Rabbit polyclonal anti-Ctip2 | Abcam | Cat# ab18465,  RRID: AB_2064130 |
| Rabbit polyclonal anti-Foxp2 | Abcam | Cat# ab16046,  RRID: AB_2107107 |
| Rabbit polyclonal anti-Pax6 | Abcam | Cat# ab195045, RRID: AB_2750924 |
| Rabbit polyclonal anti-Nanog | Cell Signaling Technology | Cat# 4903,  RRID: AB_10559205 |
| Rabbit polyclonal anti-Sox2 | Abcam | Cat# ab93689, RRID: AB_10562630 |
| Rabbit polyclonal anti-Ki67 | Abcam | Cat# ab15580, RRID: AB_443209 |
| Mouse polyclonal anti-Brn2 | Millipore | Cat# MABD51, RRID: AB_11204531 |
| Mouse polyclonal anti-Stab2 | Abcam | Cat# ab51502, RRID: AB_882455 |
| Rabbit polyclonal anti-Hopx | (Santa Cruz Biotechnology | Cat# sc-30216,  RRID: AB_2120833 |
| Rabbit polyclonal anti-CD31 | Abcam | Cat# ab28364,  RRID: AB_726362 |
| Mouse monoclonal anti-human nuclei | Millipore | Cat# MAB1281, RRID: AB_94090 |
| Mouse monoclonal anti-STEM121 | Takara Bio | Cat# Y40410,  RRID: AB_2801314 |
| **Chemicals** |  |  |
| Hoechst 33342 | Invitrogen | Cat# H1399 |
| NaCl | Biosharp, China | Cat# BL542A |
| Neural induction medium | Thermo Fisher | Cat# A1647801 |
| N2 | Thermo Fisher | Cat# 17502001 |
| DMEM/F12 | Thermo Fisher | Cat# 21331020 |
| MEM-NEAA | Thermo Fisher | Cat# 10370021 |

**The source, Cat# number and RRID of all antibodies and chemicals used are shown.**
